# Advances in Epicardial Biology: Insights from Development, Regeneration, and Human Cardiac Organoids

**DOI:** 10.3390/jcdd12100389

**Published:** 2025-10-02

**Authors:** Shasha Lyu, Alvin Gea Chen Yao, Yu Xia, Jingli Cao

**Affiliations:** 1 Cardiovascular Research Institute, Weill Cornell Medicine, 1300 York Avenue, New York, NY 10021, USA; shl4037@med.cornell.edu (S.L.); aly4010@med.cornell.edu (A.G.C.Y.); yux4005@med.cornell.edu (Y.X.); 2 Department of Cell and Developmental Biology, Weill Cornell Medicine, 1300 York Avenue, New York, NY 10021, USA

**Keywords:** epicardial cells, heart development, cardiac regeneration, epicardial-derived cells (EPDCs), cardiac organoids

## Abstract

The epicardium plays a pivotal role in heart development, regeneration, and disease response through its contributions to multiple cardiac lineages and its dynamic paracrine signaling. Recent advances in lineage tracing, single-cell technologies, and, particularly, human pluripotent stem cell (hPSC)-derived cardiac organoid models have illuminated the cellular heterogeneity, developmental plasticity, and intercellular crosstalk of epicardial cells with other cardiac cell types. These models have revealed conserved and divergent mechanisms of epicardial function across species, offering new insights into epicardial–myocardial–endothelial–immune interactions and the regulation of cardiac repair. This review highlights recent key findings from developmental and regenerative studies, integrating them with emerging data from human cardiac organoids to provide an updated framework for understanding epicardial biology and its therapeutic potential.

## 1. Introduction

The epicardium, a mesothelial layer enveloping the heart, is highly conserved in both structure and function across vertebrates, including zebrafish and mammals [1,2,3]. During vertebrate heart development, the pro-epicardium gives rise to the epicardial cell layer that spreads over the myocardium to form the epicardium [3,4,5]. Beyond serving as a protective outer layer, the epicardium plays a crucial role in cardiac development and holds significant potential in regenerative medicine. During embryogenesis, epicardial cells undergo epithelial-to-mesenchymal transition (EMT), giving rise to epicardial-derived cells (EPDCs). These EPDCs contribute to multiple cardiac lineages, including fibroblasts, vascular smooth muscle cells (vSMCs), pericytes, and adipocytes [2,3]. Additionally, the epicardium secretes paracrine factors, such as retinoic acid (RA) and fibroblast growth factors (FGFs), which are essential for myocardial proliferation and coronary vessel formation [2,3].

In the adult heart, the epicardium is largely quiescent under homeostatic conditions but can be reactivated in response to injury. Comparative studies have shown that species such as zebrafish exhibit robust epicardial-mediated heart regeneration, whereas adult mammals display limited regenerative capacity. Following cardiac injury in zebrafish, the epicardium reactivates embryonic gene programs and produces key paracrine signals (e.g., neuregulin 1 [Nrg1] and vascular endothelial growth factor Aa [vegfaa]) and extracellular matrix (ECM) components that support cardiomyocyte (CM) proliferation and coronary angiogenesis. The epicardium also serves as a source of vSMCs, pericytes, and fibroblasts, and is essential for successful heart regeneration in zebrafish [6,7,8,9,10,11,12]. In contrast, although the adult mammalian epicardium undergoes similar activation following injury, it fails to produce sufficient mitogens or differentiation signals to support regeneration [13,14,15,16,17]. Notably, delivering epicardial-derived factors or transplanting stem cell-derived epicardial cells has been shown to improve cardiac function in mammalian myocardial infarction models [14,18]. Understanding the molecular and cellular mechanisms underlying epicardial secretion and differentiation could inform strategies to reactivate the epicardium and enhance heart repair in mammals.

While animal models such as zebrafish and mouse continue to provide valuable insights into epicardial biology, recent advances in human cardiac organoid technology have opened new avenues for in vitro modeling. Organoids derived from hPSCs have been engineered to include epicardial layers using approaches such as WNT signaling modulation and co-culture with proepicardial-like cells [19,20,21]. These models recapitulate key features of heart development, including epicardial EMT, myocardial maturation, and vascularization. Epicardial cells within cardiac organoids have been shown to influence CM compaction and contribute to vascular structure formation, underscoring their functional importance. In this review, we summarize recent advancements in epicardial biology, with a focus on insights gained from developmental and regenerative studies in animal models, as well as emerging findings from human cardiac organoid studies.

## 2. Modeling Epicardial Biology Using Human Cardiac Organoids

### 2.1. Advances in Cardiac Organoid Technology

hPSC-derived cardiac organoids have emerged as transformative platforms for studying heart development, disease mechanisms, and regenerative biology in vitro. Over the past decade, advances in stem cell differentiation, bioengineering, and tissue patterning have progressively increased the complexity and physiological relevance of these models, enabling the exploration of epicardial development and epicardium-mediated cell–cell interactions in a human context. Early cardiac organoids primarily focused on recapitulating myocardial features such as contractility, CM maturation, and primitive chamber formation but did not fully reflect the processes of early heart development [22,23,24].

A significant step forward came with the study by Drakhlis et al., who established self-organizing cardiac organoids capable of modeling early cardiogenesis. By embedding hPSC aggregates in matrigel and inducing cardiac differentiation via WNT signaling modulation, they generated 3-dimensional (3D) structures resembling embryonic heart development [25]. These organoids contained myocardial and endocardial layers, septum transversum-like mesoderm, vascular networks, and spatially distinct foregut endoderm. Remarkably, *NKX2.5*-knockout organoids exhibited malformations analogous to congenital heart defects observed in mice, demonstrating the utility of this system for modeling genetic cardiac diseases. Complementing these findings, Rossi et al. developed an embryonic organoid system in mice using axially patterned mouse embryonic stem cells (mESCs)-derived gastruloids. These structures recapitulate the spatiotemporal events of early heart organogenesis with remarkable fidelity, generating cardiovascular progenitors from both the first and second heart fields. These progenitors self-organize into a cardiac crescent-like structure, which matures into beating cardiac tissue positioned adjacent to a gut-like tube and separated by an endocardial-like layer. This model underscores the intrinsic morphogenetic potential of pluripotent cells and provides a robust platform for dissecting early cardiovascular development in vitro [26].

**Figure 1:**
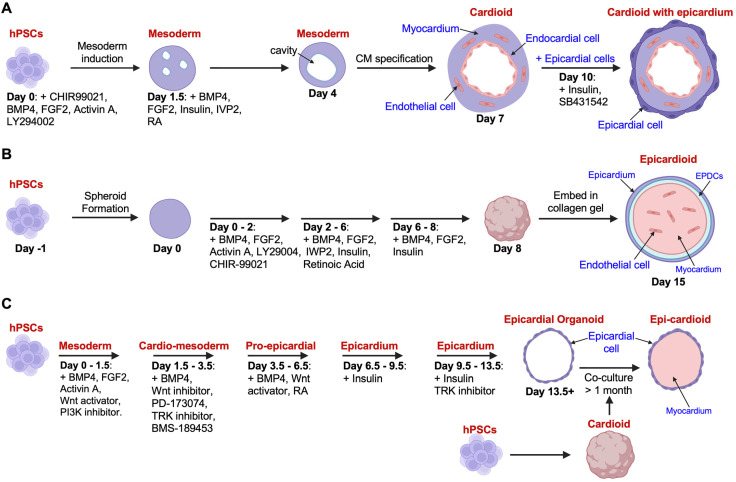
Overview of cardiac organoid generation from hPSCs. Three recently published protocols are illustrated. (**A**) Chamber-like cardioids containing epicardial, myocardial, and endocardial layers [21]. (**B**) Self-assembled epicardioids composed of epicardium, EPDC, myocardium, and endothelial cells [19]. (**C**) Epicardial cell-only organoids fused with cardioids to form “Epi-cardioid”, which contain both epicardial and myocardial layers [27]. Created in BioRender. Yao, A. (2025) https://BioRender.com/v27c6o9.

A breakthrough in modeling epicardial biology came from Hofbauer et al., who created the first epicardium-inclusive cardiac organoids termed “cardioids” (Figure 1A) [21]. These hPSC-derived cardioids self-organize to form chamber-like structures with central cavities, mimicking aspects of early heart chamber morphogenesis. WNT-BMP signaling and the downstream effector HAND1 were shown to be essential for cavity formation, linking cardioid development to pathways implicated in congenital heart defects. Furthermore, using mesodermal patterning protocols allowed the generation of epicardial progenitors that were integrated into cardioids either through co-differentiation or stepwise assembly. Notably, these epicardium-inclusive cardioids responded to cryoinjury with fibrotic-like ECM remodeling, establishing their value for studying injury responses and regenerative signaling. However, the separate generation of epicardial cells reduces the relevance of this model for studying epicardial-myocardial co-development.

Similarly, Branco et al. introduced a multilineage human pro-epicardium/foregut organoid system [20]. By first differentiating hPSCs into pro-epicardium, septum transversum mesenchyme, and foregut/liver bud lineage aggregates via modulation of WNT, BMP, and retinoic acid (RA) signaling, the researchers then co-aggregated these with CM spheroids at a defined ratio. This resulted in a self-organizing heart organoid where a WT1^+^ epicardial-like layer fully envelops a TNNT2^+^ myocardium-like core, recapitulating endogenous tissue-tissue interactions observed during early heart formation and offering a tractable system for probing epicardial contributions to myocardial development and function. This represents a more complex model, as additional non-cardiac tissues are included, though it offers greater physiological relevance in mimicking in vivo development.

Most recently, Meier et al. developed “epicardioids”—hPSC-derived, self-organizing organoids that faithfully model the epicardial and myocardial architecture in the left ventricular wall in a RA-dependent manner (Figure 1B) [19]. By integrating lineage tracing, single-cell transcriptomics, and chromatin accessibility profiling, the authors mapped the specification and differentiation of epicardial and myocardial populations, drawing transcriptional parallels with human fetal heart development. Importantly, epicardioids enabled functional interrogation of epicardial-myocardial crosstalk, revealing critical roles for IGF2/IGF1R and NRP2 signaling in cardiogenesis. Furthermore, these models successfully recapitulated disease-relevant features such as hypertrophy and fibrotic remodeling, demonstrating their potential as a powerful platform for studying epicardial regulation of cardiac development, disease, and repair. However, in contrast to the Hofbauer model, the epicardioids do not typically form cavities in the center. More importantly, these epicardioids are embedded in collagen gels in the protocol, which may stimulate epicardial cells to migrate outward into the gel. While this phenomenon could resemble epicardial expansion in vivo, in the context of organoid modeling, it represents a disadvantage, as it disrupts the structural integrity of the organoid and limits the ability to study coordinated epicardial-myocardial co-development within a self-contained system.

In addition, Wang et al. generated hPSC-derived epicardial organoids consisting solely of an epicardial layer (Figure 1C) [27]. These organoids recapitulated key epicardial features, including EMT, ECM secretion, and differentiation into sSMCs. When co-cultured with hPSC-derived cardioids for over a month, they fused to form “Epi-cardioids,” which contained both epicardial and myocardial layers. Upon implantation into immunodeficient mice, epicardial-derived cells migrated into the host heart (including the left ventricular posterior wall and interventricular septum), where they co-expressed the EMT marker *SNAI1* and displayed CM-like morphology, suggesting possible epicardial-to-CM transdifferentiation, which we will further discuss in Section 3.2. Again, the co-culture method of separately generated epicardial cells and CMs reduces the relevance of this model for studying epicardial-myocardial co-development.

### 2.2. Challenges and Future Directions in Cardioid Application

Together, these progressively refined cardiac organoid models provide unprecedented access to the dynamic processes of epicardial specification, signaling, and function. They offer versatile experimental systems for mechanistic interrogation of epicardial EMT, differentiation, and interactions with myocardial and endothelial lineages, which are detailed in Section 3 and Section 4. Additionally, epicardioids present a versatile platform for high-throughput drug screening and toxicity testing, particularly for compounds that modulate tissue repair pathways.

Nonetheless, continued refinement is needed to fully harness the potential of epicardium-inclusive cardioids. First, there is a pressing need to refine differentiation protocols to more faithfully replicate the cellular diversity of the heart including epicardial, myocardial, vascular, immune, and even neural lineages. Integrating these cellular components guided by developmental cues from animal models will enhance physiological relevance and modeling of tissue-level interactions [28]. Efforts to co-culture or co-differentiate immune cells, such as macrophages, are underway to better model inflammatory and fibrotic responses—both critical to epicardial biology [29,30,31,32]. Also, co-differentiation of key cardiac cell types (i.e., CMs, epicardial cells, and endothelial cells) would more faithfully recapitulate the coordinated developmental processes observed in vivo.

Second, variability in cardioid generation protocols between laboratories remains a major obstacle. Differences in extracellular matrices, morphogen dosing, and scaffolding contribute to inconsistencies. The field would benefit from standardized protocols, consistent use of single-cell and spatial transcriptomic assays, and robust quality control metrics to ensure reproducibility.

Third, current organoid systems retain a fetal-like epicardial state, which constrains their utility for modeling adult disease. Methods to promote maturation are needed, and recent progress using mTOR inhibition has shown promise in inducing quiescence in human induced PSC (hiPSC)-derived epicardial cells, thereby better mimicking mature epicardium [33].

Lastly, emerging engineering approaches, such as embedding electrically conductive nanowires, have shown promise in enhancing electromechanical properties and therapeutic efficacy of cardiac organoids in rodent infarct models [34]. These refinements will position epicardial organoids as powerful tools for uncovering fundamental mechanisms of epicardial biology and advancing translational regenerative therapies for heart diseases.

## 3. The Epicardial Lineage

### 3.1. Cellular Heterogeneity and EMT

The advances in cardioids demonstrate how organoid systems are becoming increasingly sophisticated tools for dissecting epicardial biology. Yet, to fully capitalize on these models, it is critical to understand the intrinsic heterogeneity of the epicardium itself and its diverse lineage contributions. Following EMT triggered by signaling pathways such as TGFβ, PDGF, and WNT, epicardial progenitors differentiate into multiple cell types, including fibroblasts, vSMCs, pericytes, and adipocytes [3,5,35,36,37]. However, the full extent of cellular heterogeneity and the regulatory networks governing epicardial EMT and lineage diversification remain incompletely understood (reviewed previously in [1,2,38,39] and summarized in Table 1). Recent advances have highlighted epicardial cellular heterogeneity, which likely underlies the generation of diverse epicardial-derived progenies. Significant epicardial heterogeneity in the adult zebrafish was first highlighted using single-cell RNA sequencing (scRNA-seq), which identified at least three distinct epicardial subpopulations [40]. Later, Weinberger et al. combined scRNA-seq, lineage tracing, and genetic tools to characterize transcriptional and functional diversity in the developing zebrafish epicardium. They defined three epicardial subpopulations: *tgm2b^+^* cells regulating epicardial expansion, *sema3fb^+^* cells limiting *tbx18^+^* epicardial cells in the outflow tract, and *cxcl12a^+^* cells recruiting *ptprc*/CD45^+^ myeloid cells to the heart [41]. In the regenerating adult zebrafish heart, recent studies have identified a transiently activated epicardial progenitor cell (aEPC) population marked by *ptx3a* and *col12a1b* [42]. After injury, these aEPCs emerge from the epithelial epicardium, migrate to the injury site, undergo EMT, and give rise to *pdgfrb^+^* mural cells and *hapln1a^+^* mesenchymal epicardial cells-both essential for CM proliferation and coronary angiogenesis during development and regeneration [42,43,44]. Shin et al. further revealed that *il11a*, a zebrafish homolog of interleukin-11, promotes heart regeneration by stimulating CM proliferation, coronary expansion, and epicardial activation [45]. Notably, *il11a* induction in uninjured hearts activates aEPCs that later differentiate into cardiac fibroblasts. However, prolonged *il11a* expression leads to excess fibroblast accumulation and fibrosis, driven by ERK signaling. Co-treatment with an ERK inhibitor mitigated fibrosis while preserving regenerative benefits, suggesting a potential therapeutic approach for balanced cardiac repair [45].

In the adult mouse heart, Hesse et al. used scRNA-seq and lineage tracing to profile epicardial stromal cells (EpiSCs) following myocardial infarction [46]. They identified 11 transcriptionally distinct populations organized into three main classes, two of which expressed cardiac lineage and sarcomere genes, suggesting cardiomyogenic potential. Enrichment of HIF1a-related transcripts pointed to a hypoxic epicardial niche [46]. Cross-species comparisons with zebrafish epicardial data suggest that mouse epicardial cells have reduced EMT and differentiation capacity after injury, potentially contributing to their limited regenerative response [42].

To dissect the molecular regulation of epicardial EMT, Jackson-Weaver et al. identified PRMT1 as a key regulator of epicardial lineage differentiation in the developing mouse heart [47]. Epicardial-specific *Prmt1* deletion reduced matrix and ribosomal gene expression and impaired EMT by stabilizing p53 through alternative splicing of *Mdm4*. Elevated p53 levels destabilized Slug, ultimately disrupting the formation of epicardial-derived fibroblasts, vSMCs, and pericytes, and leading to defective ventricular morphogenesis and coronary vessel formation. Astanina et al. subsequently identified TFEB as a negative regulator of EMT in embryonic mouse epicardium [48]. TFEB promotes TGIF1, a TGFβ/Smad pathway repressor, and its overexpression impairs EMT and heart development, while its loss sensitizes cells to TGFβ-induced EMT. More recently, Streef et al. used scRNA-seq to analyze the human fetal epicardium and identified CRIP1 as a previously unrecognized regulator of EMT, adding a new layer to our understanding of human epicardial biology [49].

Collectively, these studies underscore that epicardial cells are highly heterogeneous, yet how this diversity translates into lineage potential remains unresolved. A major open question is whether epicardial subtypes represent predetermined progenitors with fixed fates, or whether some populations retain broader multipotency and the capacity to generate multiple lineages (such as the transiently activated aEPCs identified in zebrafish [42]). Moreover, it is unclear how signaling pathways and microenvironmental cues dynamically regulate transitions between epicardial states across development, regeneration, and disease. Addressing these questions with integrated lineage tracing, perturbation-based single-cell approaches, and organoid models will be critical for uncovering the principles that govern epicardial plasticity and for leveraging this knowledge toward regenerative therapies.

**Table 1:** Epicardial cell fates in heart development and regeneration.

Consistent Conclusions			
Cell Fates	Species (Context)	Fate Mapping Approaches and Reagents	References
Fibroblast	Chick (Development)	Dye labelling, retroviral labelling, and cell transplantation	[50,51,52,53,54,55]
	Zebrafish (Post injury)	Transplantation of *wt1^+^* cells	[11]
	Mouse (Development)	*Tbx18^Cre^; R26R^lacZ^*	[56]
		*Tbx18^Cre^; Rosa26^mT/mG^*	[57]
		*Wt1^GFPCre^; R26R^mT/mG^*	[58]
		*Tcf21^iCre^*; *R26R^YFP^* or *R26R^tdT^*	[59]
		*Scx^GFPCre^; R26R^lacZ^* *Sema3D^GFPCre^; R26R^lacZ^*	[60]
	Mouse (Post injury)	*Wt1^CreERT2^; R26R^mT/mG^*	[13]
		*Wt1^GFPCre^; R26R*	[61]
		Thymosin β4 treatment *Wt1^CreERT2^; R26R^mT/mG^*	[62]
	Human (in vitro)	Culture of epicardial-like cells derived from hiPSCs (TGFβ1 and BFGF Treatment)	[63]
		Culture of primary epicardial cells from human adults (spontaneous differentiation)	[64]
		Culture of epicardial cells derived from H13 hESCs (TGFβ1 and BFGF Treatment)	[65]
Smooth muscle cell (SMC)	Chick (Development)	Dye labelling, retroviral labelling, and cell transplantation	[50,51,52,53,54,55]
	Mouse (Development)	*Wt1^Cre^; Rosa26^fsLz^* or Z/Red *Wt1^CreERT2^; Rosa26^fsLz^* or Z/Red	[66]
		*Tbx18^Cre^; R26R^lacZ^*	[56]
		*Tbx18^Cre^; Rosa26^mT/mG^*	[57]
		*Scx^GFPCre^; R26R^lacZ^* *Sema3D^GFPCre^; R26R^lacZ^*	[60]
	Mouse (Post Injury)	*Wt1^CreERT2^; R26R^mT/mG^*	[13]
		*Wt1^GFPCre^; R26R*	[61]
		Thymosin β4 treatment *Wt1^CreERT2^; R26R^mT/mG^*	[62]
		VEGFA modRNA treatment *Wt1^CreERT2^; R26R^mT/mG^*	[67]
	Human (in vitro)	Culture of epicardial-like cells derived from hiPSCs (TGFβ1 and BFGF Treatment)	[63]
		Culture of primary epicardial cells from human adults (TGFβ1 or BMP2 Treatment)	[64]
		Culture of epicardial cells derived from H13 hESCs (TGFβ1 and BFGF Treatment)	[65]
		Culture of hPSC-derived epicardial cells (PDGF-BB and TGFβ1 treatment)	[68]
Pericyte	Mouse (Development)	*Tbx18^Cre^; R26R^lacZ^*	[56]
	Mouse (Post injury)	*Wt1^CreERT2^; R26R^mT/mG^*	[13]
		*Wt1^CreERT2^; R26R^YFP^*	[69]
	Human (in vitro)	Culture of hiPSC-derived epicardial cells (SMAD3 promotes pericyte specification)	[70]
Perivascular cell (pericyte or SMC, not specified)	Zebrafish (Development)	*tcf21:CreER; gata5:RnG*	[6]
	Zebrafish (Post Injury)	*tcf21:CreER; gata5:RnG*	[6]
		Transplantation of *wt1^+^* cells	[11]
		*ptx3^CreERt2^; ubi:Switch*	[42]
Adipocyte	Zebrafish (Development)	*tcf21:CreER; ubi:Switch*	[71]
	Mouse (Development)	*Tbx18^Cre^; R26R^YFP^*	[72]
	Mouse (Post Injury)	*Wt1^CreERT2^; R26R^mT/mG^* or *R26R^RFP^*	[73]
		*Wt1^CreERT2^; R26R^tdT^*	[74]
**Inconsistent Findings**			
**Cell Fates (Notes)**	**Species**	**Fate Mapping Approaches and Reagents (Notes)**	**References**
Cardiomyocyte (not observed in zebrafish studies)	Mouse (Development)	*Wt1^Cre^; Rosa26^fsLz^* or Z/Red *Wt1^CreERT2^; Rosa26^fsLz^* or Z/Red (The Wt1 transgenic lines are not epicardial cell-specific)	[66]
		*Tbx18^Cre^; R26R^lacZ^* (The Cre line is not epicardial cell-specific)	[56]
		*Scx^GFPCre^; R26R^lacZ^* *Sema3D^GFPCre^; R26R^lacZ^* (Very rare)	[60]
	Mouse (Post injury)	*Wt1^GFPCre^; R26R*	[61]
		*Wt1^CreERT2^; R26R^mT/mG^* (only upon thymosin β4 treatment)	[62]
	Salamanders (Post injury)	Microinjection of Cre recombinase to label epicardial cells (Labeling specificity could affect the conclusion)	[75]
	Human (in vitro)	Culture of hPSC-derived cardioid organoid	[27]
Endocardial cell (There are limited studies. No mention of this fate in other studies.)	Chick (Development)	Dye labelling, retroviral labelling, and cell transplantation	[51,53,54]
	Mouse (Development)	*Scx^GFPCre^; R26R^lacZ^* *Sema3D^GFPCre^; R26R^lacZ^*	[60]
Endothelial cell (Has not been reported in zebrafish)	Chick (Development)	Dye labelling, retroviral labelling, and cell transplantation	[50,52,53,54,55]
	Mouse (Development)	*Wt1^Cre^; Rosa26^fsLz^* or Z/Red *Wt1^CreERT2^; Rosa26^fsLz^* or Z/Red (The Cre or CreER line is not epicardial cell-specific)	[66]
		*Scx^GFPCre^; R26R^lacZ^* *Sema3D^GFPCre^; R26R^lacZ^* (Tracing of pro-epicardial cells)	[60]
	Mouse (Post Injury)	*Wt1^GFPCre^; R26R*	[61]
		VEGFA modRNA treatment *Wt1^CreERT2^; R26R^mT/mG^*	[67]
	Human (in vitro)	Culture of epicardial cells derived from hPSCs (VEGF Treatment)	[76]

### 3.2. Contribution to Cardiomyocytes

A long-standing question in epicardial biology is whether epicardial cells can directly differentiate into CMs, a controversy that has largely arisen from differences in experimental approaches. Although early fate-mapping experiments using mouse models appeared to support epicardial contribution to CMs [56,66], later studies revealed that the commonly used *Tbx18*- and *Wt1*-based tracing tools also marked non-epicardial populations, including CMs themselves [77,78]. In addition, using *Scx*- and *Sema3d*-based Cre lines to label pro-epicardial cells, Katz et al. detected a CM fate of these labeled cells, although it is very rare [60]. However, the expression domains of *Scx* and *Sema3d* are largely distinct from those of classic epicardial markers such as *Tbx18* and *Wt1*, and these transgenic lines label only subsets of pro-epicardial cells. Whether *Scx* and *Sema3d* expression is specific to the epicardial lineage therefore remains an open question and warrants further investigation. Overall, lineage-tracing studies in both zebrafish and mice indicate that epicardial-to-CM differentiation is minimal, if it occurs at all [6,11,17,60,77]. In contrast, a recent study in salamanders demonstrated that CLDN6^+^ epicardium-derived cells appear at injury sites, undergo transcriptional reprogramming, and give rise to de novo CMs [75]. This work employed microinjection of a cell-permeant Cre recombinase with a CAG:loxP-Cherry-loxP-H2B-YFP reporter, but the specificity of epicardial labeling remains a critical caveat requiring further validation.

While more refined lineage-tracing and single-cell approaches are needed to resolve this controversy, human cardioid models provide additional support. As discussed in Section 2.1, Wang et al. demonstrated that hPSC-derived epicardial organoids (Figure 1C) transplanted into immunodeficient mice gave rise to CM-like cells, consistent with epicardial-to-CM trans-differentiation [27]. These findings suggest that epicardial cells may retain latent CM potential that can be revealed under certain developmental or injury contexts. Supporting this idea, the Riley group reported that thymosin β4 treatment prior to cardiac injury induced low-level epicardial-to-CM differentiation in mice, raising the possibility that this plasticity can be pharmacologically activated [79]. Harnessing such latent potential through post-injury reprogramming strategies could offer new avenues for cardiac repair.

### 3.3. Contribution to Non-Cardiomyocytes

While the potential of epicardial cells to generate cardiomyocytes remains debated, their contribution to non-cardiomyocyte lineages is well established and constitutes the major role of the epicardium during heart development and repair. Cardiac fibroblasts play essential roles in heart development and are key contributors to both disease progression and cardiac regeneration [80]. Across various animal models, epicardial cells have been consistently identified as the primary source of cardiac fibroblasts and also contribute to vSMCs and pericytes, supporting the formation of the coronary vasculature (summarized in Table 1) [2,3,39,81,82]. Recent advances revealed new regulators of epicardial cell EMT and differentiation to these cell fates. As mentioned above, epicardial-specific *Prmt1* deletion in mice impaired EMT and the formation of epicardial-derived fibroblasts, vSMCs, and pericytes [47]. Recently, Junghof et al. modeled human epicardial development using hiPSCs and identified CDH18, a type II cadherin, as a key marker of active epicardial identity. Loss of CDH18 triggered EMT and promoted differentiation toward SMCs [83]. GATA4 was found to regulate CDH18 expression, providing mechanistic insight into epicardial lineage commitment. Cardiac pericytes, identified by their expression of PDGFRβ and chondroitin sulfate proteoglycan 4 (CSPG4, also known as neuron-glial antigen 2 (NG2)), are essential for stabilizing the embryonic coronary plexus [39,84]. Miyoshi et al. discovered that SMAD3 promotes the specification of hiPSC-derived epicardial cells into cardiac pericyte progenitors, independently of TGFβ signaling. Loss of SMAD3 led to elevated expression of pericyte markers including endoglin (CD105), CSPG4, CD248, and CD13 [70].

Epicardial cells also contribute to cardiac adipocytes during development and after injury [72,73,74]. A recent study identified a zebrafish epicardial fat depot with molecular characteristics of beige adipocytes, a subset of which demonstrated primitive thermogenic potential [71]. Lineage tracing using a *tcf21:CreER* line showed that epicardial cells contribute approximately 72% of adipocytes in this epicardial adipose tissue. This study confirmed the conserved adipocyte fate of epicardial cells from zebrafish to mammals.

Finally, the contribution of epicardial cells to cardiac endothelial and endocardial lineages remains debated. Early studies in chick and quail using dye labeling, retroviral labeling, and cell transplantation suggested that epicardial cells can give rise to both endocardial and coronary endothelial cells [51,53,54], but these classic approaches lacked molecular resolution to define cell identities precisely. In zebrafish, no epicardial contribution to endocardial or endothelial lineages has been reported. In mouse, Katz et al. found that *Scx*- and *Sema3D*-expressing pro-epicardial cells contribute to coronary vascular endothelium, as well as to the early sinus venosus and endocardium [60]. However, as mentioned above, the lineage specificity of these transgenic tools requires further validation. Carmona et al. showed that the widely used *Wt1^GFP^* reporter is also expressed in embryonic ventricular endothelium [85]. They further use 4 transgenic tools (*Wt1^Cre^*, *Gata5^Cre^*, G2-Gata4 enhancer, and *cTnT^Cre^* lines) to demonstrate that epicardial cells contribute minimally (~4%) to the coronary endothelium. By contrast, coronary arterial endothelial cells largely derive from sinus venosus endothelial cells, which sprout into the subepicardial space and expand to form coronary arteries [86]. Most recently, using a *TBX5^Clover2^/NKX2-5^TagRFP^* reporter system to trace cardiac lineages, Zhang et al. showed that hiPSC-derived *TBX5^+^NKX2-5^−^* cells display epicardial characteristics, while *TBX5^−^NKX2-5^−^* cells resemble endothelial cells [87]. Whether these two populations diverge from a common progenitor or follow separate differentiation trajectories remains to be further investigated. Collectively, these findings highlight the limitations of relying on single lineage-tracing tools, emphasizing the need for dual recombinase systems [88] and single-cell approaches to clarify the epicardial contribution to endothelial and endocardial lineages.

Overall, the epicardium exhibits striking plasticity, giving rise to diverse lineages depending on developmental stage, injury context, and signaling environment. A key unresolved challenge is how to direct its differentiation toward pro-regenerative fates (e.g., CMs or endothelial cells) rather than fibrotic lineages. Elucidating the mechanisms that govern these choices will be essential for advancing our understanding of heart biology and for developing strategies to harness epicardial cells in cardiac repair.

## 4. Paracrine Signaling and Cellular Crosstalk

**Figure 2:**
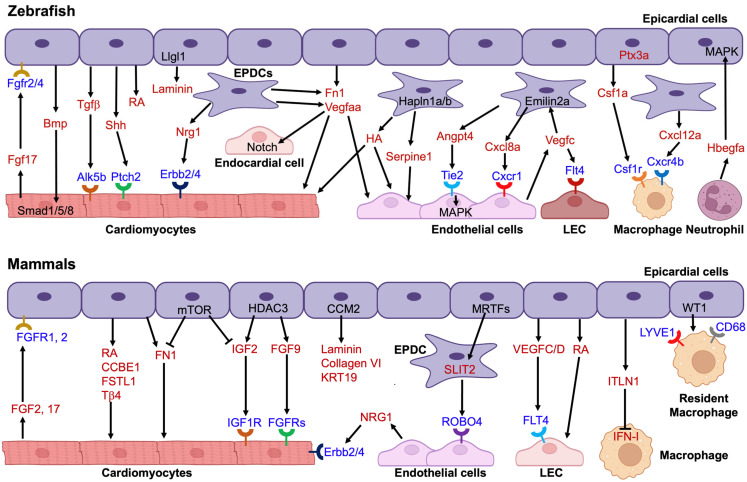
Updated molecular interactions among epicardial cells and other cardiac cell types during heart development and regeneration. Paracrine signaling from epicardial cells or EPDCs mediates crosstalk between them and other cardiac lineages during development and regeneration in zebrafish (**up**) and mammals (**bottom**). Red font indicates secreted proteins, while blue font indicates receptors. Besides the recently identified molecular interactions referenced in the main text, other factors were reviewed previously. LEC, lymphatic endothelial cell; RA, retinoic acid; FN1, fibronectin 1; HA, hyaluronic acid; Nrg1, neuregulin 1; Tβ4, thymosin beta 4; ITLN1, intelectin1. Created in BioRender. Yao, A. (2025) https://BioRender.com/pfndie2.

The cellular diversity underpins the remarkable signaling capacity of epicardium, shaping not only its own fate decisions but also influencing CMs, endothelial cells, and immune populations. The epicardium and its derivatives serve as a dynamic signaling hub that orchestrates CM proliferation and coronary vessel formation during both cardiac development and regeneration [1,3,39]. Epicardial cells secrete a diverse array of signaling and structural molecules, including RA, FGFs, insulin-like growth factor 2 (IGF2), Fibronectin (Fn), Shh, Neuregulin 1 (Nrg1), BMP, WNT, and Follistatin-like 1 (Fstl1), which influence myocardial growth, ECM remodeling, and tissue repair (reviewed previously in [1,2,38,39] and summarized in Figure 2). Recent studies using animal models and epicardial-inclusive cardiac organoids have significantly advanced our understanding of epicardial paracrine signaling and intercellular interactions, uncovering new cellular and molecular interactions (Figure 2). Bonet et al. demonstrated that CCBE1, an extracellular matrix protein secreted by epicardial cells, is indispensable for epicardial expansion and myocardial communication in mice. Loss of *Ccbe1* disrupts both epicardial and CM proliferation, impairs epicardial cell migration, and reduces EPDC contribution to the heart, likely via deregulation of EMT-related genes [89]. In both zebrafish and human models, oxytocin, a hypothalamic neuropeptide, emerged as a regulator of epicardial function. Oxytocin promotes epicardial proliferation, EMT, and gene expression via TGFβ signaling, and its inhibition impairs both embryonic epicardial development and adult regeneration [90]. These findings suggest that central neuroendocrine signals can influence cardiac regeneration through epicardial reprogramming. Most recently, Wang et al. revealed that epicardial-specific deletion of *Ccm2* in mice leads to defects in epicardial adhesion, polarity, migration, and spreading. These disruptions result in impaired heart development and delayed recovery after myocardial infarction. Mechanistically, CCM2 modulates the expression of cytoskeletal and matrix proteins, including KRT19, Laminin, and Collagen VI, underscoring its pivotal role in maintaining epicardial architecture and function [91].

### 4.1. Epicardial-Endothelial Interactions

The epicardium also plays a key role in guiding coronary and lymphatic vascular development through interactions with endothelial cells (ECs; previous studies are summarized in Figure 2). Here we provide recent updates on this interaction. Quijada et al. identified a subset of *Slit2^+^* EPDCs that emerge post-EMT and function as guidepost cells to control angiogenesis and vascular stability through Slit2-Robo4 interactions. Epicardial-specific deletion of myocardin-related transcription factors (MRTFs) impaired guidance cue expression, endothelial-EPDC interactions, and led to persistent immature angiogenic ECs [92]. Further emphasizing the epicardial role in vascular guidance, Sun et al. identified *hapln1a^+^* EPDCs in juvenile zebrafish that form HA-rich linear scaffolds guiding coronary vessel growth. These cells also express *serpine1*, driving coronary angiogenesis. Disruption of either *hapln1a* or *serpine1* abrogates proper vascularization [43].

Expanding this framework, Wu et al. used scRNA-seq to uncover a regeneration-induced EPDC population in zebrafish that transiently expresses *angpt4*. This signal activates Tie2-MAPK signaling in the endocardium, subsequently triggering RA signaling in CMs to promote proliferation and scar resolution [93]. In humans, Knight-Schrijver et al. compared fetal and adult epicardium at single-cell resolution, revealing that fetal epicardium contains migratory, angiogenic fibroblast-like cells, while adult epicardium exhibits mesothelial and immune-responsiveness. Fetal-specific WNT signaling between epicardium and endocardium may underlie the enhanced regenerative capacity seen during development [94].

The epicardium also plays an essential paracrine role in cardiac lymphatic development and function. While most cardiac lymphatic endothelial cells (LECs) derive from venous origins [95,96], epicardial-derived signals are indispensable for their growth and spatial distribution. Lineage tracing and expression analyses in avian and mouse embryos revealed the early appearance of lymphatic markers such as PROX1, LYVE-1, and VEGFR-3 within the epicardial region, supporting its involvement in lymphatic development [97]. In addition to paracrine signaling, EPDCs and their interactions with the second heart field (SHF) contribute to the patterning and expansion of cardiac lymphatic vasculature in mice [98]. In this study, Lioux et al. showed that the epicardium and arterial mesothelial cells establish a vasculogenic niche at the base of the great arteries, partly derived from SHF progenitors. Within this niche, sub-mesothelial cells differentiate into LECs, which, together with mesothelial derivatives, are essential for ventral cardiac lymphatic development. Regional retinoic acid signaling from epicardial cells further regulates this process, suggesting both structural and molecular support of the epicardium in cardiac lymphangiogenesis [98]. Using single-nucleus multiomic analyses, Travisano et al. identified distinct populations of epicardial-resident LECs in human fetal hearts and showed that VEGFC is highly expressed in arterial endothelial cells and EPDCs, providing a molecular basis for the arterial association of cardiac lymphatics [99]. More recently, de la Cruz et al. demonstrated that VEGFC produced by embryonic epicardial cells is critical for cardiac lymphangiogenesis in mice, with VEGFD acting cooperatively in a female-specific manner. Epicardial deletion of these ligands severely impaired lymphatic formation, highlighting the essential role of VEGFC/D-mediated signaling in epicardial regulation of cardiac lymphatic development [100].

As in humans, zebrafish cardiac lymphatics track along coronary arteries [99,101,102]. However, in zebrafish, *vegfc* expression is minimal in the epicardium during both heart development and regeneration [101]. Instead, it is upregulated in regenerating coronaries post-injury [103]. El-Sammak et al. showed that Vegfc promotes endothelial proliferation and induces *emilin2a* expression in EPDCs, which in turn triggers *cxcl8a* expression, activating Cxcl8a-Cxcr1 signaling to support revascularization and CM regeneration. These findings establish a zebrafish-specific Vegfc-Emilin2a-Cxcl8a/Cxcr1 axis in epicardial-endothelial signaling during regeneration. Potential epicardial expression and function of *vegfd* warrant further investigation.

These studies highlight the critical instructive role of the epicardium in guiding endothelial behavior, but key questions remain about the mechanisms that define epicardial subpopulation identity and their interaction with endothelial cells. Determining whether specific EPDC subsets possess intrinsic angiogenic (e.g., *hapln1^+^* subtype) or lymphangiogenic potential, or whether these functions are induced by local cues, will be important for understanding developmental and regenerative programs.

### 4.2. Crosstalk with Cardiomyocytes

The epicardium and its derivatives have long been recognized as a critical signaling hub that regulates CM proliferation [104,105,106]. Beyond developmental roles, the epicardium secretes a diverse array of paracrine factors (including growth factors, cytokines, and extracellular vesicles) that promote CM proliferation, survival, and metabolic adaptation during regeneration (reviewed in [1,39]). Despite these advances, the precise cellular and molecular mechanisms underlying epicardial-CM crosstalk remain incompletely understood. Here, we summarize recent studies that provide new cellular and molecular insights into the dynamic interplay between the epicardium and CMs.

Jang et al. found that epicardial HDAC3 governs the secretion of FGF9 and IGF2, mitogens essential for myocardial wall development by binding to their receptors in CMs (IGF1R and FGFRs). Loss of *Hdac3* leads to myocardial hypoplasia and diminished epicardial derivatives, which are linked to the upregulation of miR-322/503 that represses *Fgf9* and *Igf2* [107]. These findings uncover a novel HDAC3-miR-322/503-FGF9/IGF2 axis, highlighting chromatin-level regulation of epicardial paracrine signaling critical for heart development and regeneration.

In zebrafish, a subset of *hapln1a/b^+^* EPDCs is critical for heart growth and regeneration. These cells emerge post-embryonically, localize near proliferating CMs, and facilitate HA deposition, supporting ventricular wall maturation and regeneration [44]. Similarly, Pollitt et al. showed that Llgl1 (Lethal giant larvae 1) coordinates epicardial development and laminin deposition in the zebrafish heart. Its absence causes premature CM extrusion and myocardial disorganization, emphasizing the importance of timely epicardial coverage for ventricular morphogenesis [108].

In hPSC-derived systems, epicardial cells promote CM function both in vitro and in vivo. Bargehr et al. demonstrated that hESC-derived epicardium supports the development of three-dimensional engineered heart tissues (3D-EHTs) in vitro and in cardiac grafts in vivo by enhancing CM maturation, proliferation and contraction. Fibronectin (FN1) emerged as a key ECM molecule mediating these effects [18]. Further, Ong et al. confirmed that epicardial FN1 acts as a mediator in the communication between epicardial cells and CMs, promoting CM maturation in 3D-EHTs. FN1 is necessary for CM maturation, as its inhibition disrupts contractility and calcium handling, while recombinant FN1 rescues these defects [109]. This aligns with zebrafish studies highlighting Fn1 as a conserved epicardial-derived paracrine factor [9].

Moreover, co-differentiation of hiPSC-derived pre-epicardial cells (PECs) and CMs leads to the consolidation of CMs into dense aggregates [110]. These aggregates form a connected beating syncytium with improved contractility and calcium handling. This study also demonstrated that PECs secrete IGF2 and stimulate CM proliferation in co-culture. Three-dimensional PEC-CM spheroid co-cultures form outer smooth muscle cell layers on cardiac microtissues with organized internal luminal structures [110]. Similarly, Meier et al. used epicardioids to show that IGF2 from epicardial cells increases CM proliferation via IGF1R in a dose-dependent manner [19]. Also, Floy et al. found that co-culture of epicardial cells and cardiac progenitors promotes CM proliferation and sarcomere organization, with TGFβ inhibition maintaining epicardial progenitors in a pro-proliferative state [111].

Most recently, two new studies using hPSCs further underscored the importance of epicardial maturity and crosstalk in orchestrating CM development and functional recovery after injury. Givens et al. demonstrated that co-culturing hiPSC-derived CMs with epicardial and EPDCs promotes CM proliferation and enhances electrophysiological and myofilament maturation, especially in 3D engineered heart tissues [112]. Tian et al. addressed the challenge of modeling adult epicardial reactivation by developing an mTOR-inhibition strategy to induce quiescence in hiPSC-derived epicardial cells, mimicking a mature, non-proliferative state [33]. These mature epicardial cells express *IGF2* and *FN1* and promote transcriptional maturation of sarcomere-related genes in hiPSC-derived CM in cardioid.

While these studies provided new insight, major gaps remain regarding how specific epicardial subpopulations are programmed (such as the aEPCs and *hapln1a^+^* epicardial subtypes [42,44]), how their signals are coordinated in time and space, and how injury or developmental context modulates their output. Future work using lineage-resolved perturbation, single-cell multi-omics, and organoid models could clarify these mechanisms and identify strategies to harness epicardial-CM crosstalk for regenerative therapy.

### 4.3. Crosstalk with Immune Cells

In addition to cardiac lineages, the epicardium modulates immune cell dynamics in the heart. Tissue-resident macrophages are the dominant immune population in the uninjured heart and primarily derive from yolk sac progenitors maintained by local proliferation [113]. While their functions in the heart have been studied, the mechanisms governing their recruitment and retention in the heart remain elusive. Stevens et al. showed that the majority of early epicardial LYVE1^+^ cells are fetal yolk macrophages expressing CD68 and located below the epicardium. Ablation of the epicardium or disruption of WT1 impairs their development, establishing the epicardium as a key niche for macrophage colonization [114].

Following injury, macrophages play crucial reparative roles, but the signals guiding their recruitment remain poorly defined. In zebrafish, Sun et al. identified *ptx3a^+^* epicardial cells expressing *csf1a* that signal to *csf1r^+^cxcr4b^+^* macrophages. Blocking this interaction impairs reparative macrophage recruitment, suggesting an epicardial niche guides beneficial immune responses [115]. Conversely, Bruton et al. found in zebrafish larval heart regeneration that macrophages stimulate epicardial *vegfaa* expression, which activates endocardial Notch signaling to promote CM proliferation, forming a macrophage-epicardium-myocardium axis for regeneration [116]. In mammalian systems, Luo et al. demonstrated that hPSC-derived epicardial cells injected into infarcted mouse hearts modulate immune responses by secreting intelectin 1 (ITLN1). ITLN1 antagonizes IFN-I signaling, promoting reparative macrophage polarization and attenuating cytokine release and leukocyte infiltration, thereby improving cardiac repair [117].

In addition to macrophages, Peterson et al. revealed that neutrophils play a critical role in initiating epicardial responses during zebrafish heart regeneration. Neutrophils rapidly mobilize to injury sites, associate with proliferating epicardial cells, and promote their expansion via Fgf and MAPK/ERK pathways, including secretion of the Hbegfa ligand. Neutrophil depletion impairs epicardial activation and myocardial regeneration, underscoring the importance of early neutrophil-epicardium interactions in heart regeneration [118].

Together, these findings illuminate the multifaceted and evolutionarily conserved roles of the epicardium in coordinating immune, endothelial, and myocardial responses through finely tuned paracrine and cellular crosstalk mechanisms.

## 5. Perspectives

Over the past decade, studies across zebrafish, mouse, and human model systems, including cardioid platforms, have substantially expanded our understanding of epicardial biology, from lineage heterogeneity and EMT to paracrine regulation of CMs, endothelial cells, and immune cells. These organoid platforms are beginning to replicate key morphogenetic and signaling events observed in vivo, offering powerful systems to dissect the mechanisms of epicardial–myocardial–endothelial–immune crosstalk with high precision.

Despite these advances, significant gaps remain. We still lack a comprehensive understanding of how specific epicardial subpopulations are programmed, how they dynamically respond to injury, and how they balance regenerative versus fibrotic outcomes. Critical questions also remain regarding their potential contribution to de novo CM formation and the signals that govern context-dependent paracrine activity. Addressing these challenges will require integrated approaches combining single-cell and spatial multi-omics, refined lineage-tracing strategies (e.g., dual recombinase systems), and next-generation multilineage organoids capable of modeling both development and disease.

Looking forward, harnessing epicardial cells therapeutically will depend on our ability to control their fate and function in a context-specific manner through reactivating developmental programs for regeneration, limiting adverse remodeling, and engineering epicardium-inclusive cardiac grafts. As human epicardial models continue to mature, they offer an unprecedented opportunity not only to unravel fundamental biology but also to translate these insights into regenerative strategies for cardiovascular disease.

## Data Availability

No new data were created or analyzed in this study. Data sharing is not applicable to this article.
